# Anterior Lumbar Interbody Fusion as a Supplement to Posterior Instrumentation in Adult Spinal Deformity Patients: A Pilot Randomized Study With a Median of Eight Years of Follow-Up

**DOI:** 10.7759/cureus.70020

**Published:** 2024-09-23

**Authors:** Lærke C Ragborg, Casper Dragsted, Dennis W Hallager, Benny Dahl, Martin Gehrchen

**Affiliations:** 1 Spine Unit, Department of Orthopedic Surgery, Rigshospitalet, Copenhagen, DNK

**Keywords:** adult spinal deformity, alif, anterior lumbar interbody fusion, asd, mechanical failure, rct, revision surgery

## Abstract

Aim

We aim to assess the long-term revision rates in patients with adult spinal deformity (ASD) undergoing posterior instrumentation with or without supplemental anterior lumbar interbody fusion (ALIF) with a median of eight years of follow-up.

Materials and methods

Based on a previous pilot randomized controlled trial (RCT) from 2012, all previous participants were invited to a clinical and radiographic follow-up. Full medical records from the total cohort were reviewed from the time of operation to the follow-up, and information on revision surgery due to mechanical failure was obtained and compared between the groups.

Results

Of the original 17 patients included in the RCT, 15 were available for follow-up and 10 attended the clinical and radiographic examination. A retrospective review was performed of the entire original cohort. The median age at follow-up was 67 (61-71) years, and the median follow-up time was 7.7 (5.1-8.8) years. Revision rates among ALIF patients were three out of seven (43%) and eight out of 10 (80%) among non-ALIF patients with pseudoarthrosis and rod breakage being the main cause. Time to failure was longer in ALIF patients with a median of 47 (28-53) months compared with non-ALIF patients with a median of 26 (9-31) months.

Conclusion

This study revealed a lower rate of revision surgery and a longer time to failure in patients undergoing ASD surgery with supplemental ALIF compared with posterior instrumentation alone. Further studies with a larger sample size are needed to make conclusions on the effect of a supplemental ALIF to posterior instrumentation on lowering the risk of mechanical failure in patients with adult spinal deformity.

## Introduction

Adult spinal deformity (ASD) is a common spinal disorder affecting overall health-related quality of life (HRQoL) due to increased pain and disability [1-3]. Abnormal curvature of the spine subsequent to degeneration can result in spinal imbalance leading to muscular fatigue, back pain, and nerve compression that can ultimately lead to a significant decrease in HRQoL [4,5]. The current surgical treatment for ASD typically consists of posterior fixation of the spine, spanning from the thoracic to the lumbosacral segments. Pseudarthrosis and implant failure comprise a substantial risk in ASD patients undergoing this type of surgery, with a high revision risk over time (>30% within five years) [6-10]. Several strategies have been suggested to improve the fusion of the instrumented segments. Supplemental anterior lumbar interbody fusion (ALIF), used at increased risk levels, is thought to improve fusion rates, stabilizing the anterior column and thus lowering the risk of mechanical failure [11,12]. However, the use of supplemental ALIF comes with an additional risk of peri- and postoperative complications, given the nature of the additional procedure and the risk of visceral and vascular injury related to the surgical approach [13,14]. A clinical benefit regarding lower revision rates should therefore commensurate with the additional risks.

This study aimed to assess long-term revision rates in patients with ASD undergoing posterior instrumentation with supplemental ALIF and compare them with patients with posterior instrumentation alone based on a previous pilot randomized controlled trial (RCT) from our institution.

## Materials and methods

Pilot RCT

**Figure 1:**
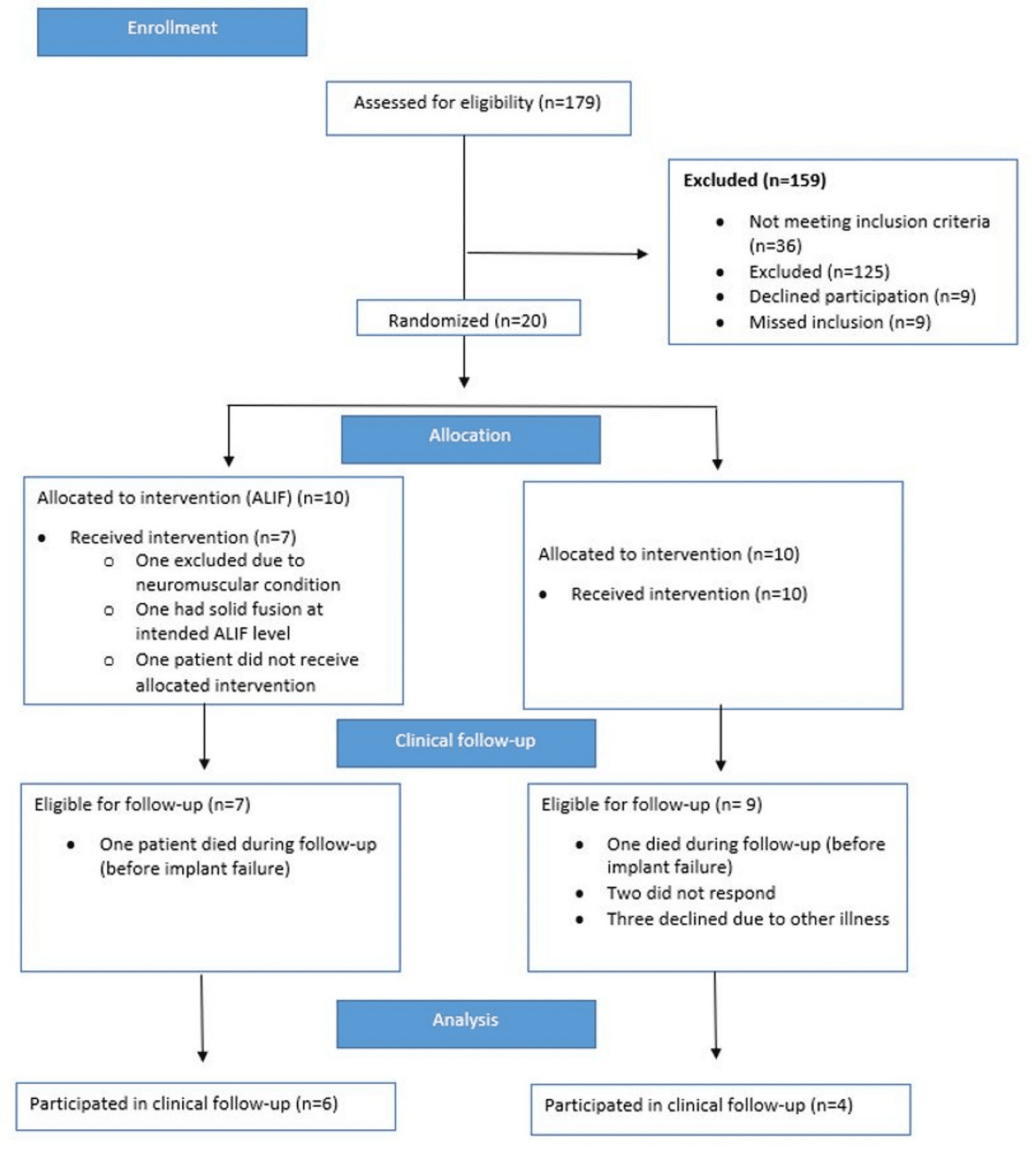
Inclusion flowchart for clinical follow-up ALIF: anterior lumbar interbody fusion

A pilot RCT was performed at our quaternary institution from December 2012 to January 2015. Patients with ASD planned for posterior instrumentation were randomized using block randomization in groups of eight to either posterior instrumented surgery or posterior instrumented surgery with supplemental ALIF at L4/L5 and/or L5/S1. The allocation to the ALIF or non-ALIF arms was enclosed in a non-transparent envelope and unblinded upon participant enrollment and acceptance. Of 179 patients assessed, 38 were eligible for inclusion. Exclusion criteria were previous ASD spine surgery, fractures, and infectious, malignant, and neuromuscular disease. Of these, nine declined participation and an additional nine were missed for inclusion, resulting in 20 patients included for randomization. Three patients in the supplemental ALIF group were later excluded; one did not receive allocated intervention, one was diagnosed with a neuromuscular disease and thus excluded, and another had solid fusion at the intended ALIF level and could not receive the allocated treatment (Figure 1). Thus, 17 patients were included in the pilot RCT. Patients were initially followed for two years postoperatively, and revision rates were compared. As the two-year results were not previously published, they are included in the following study.

Data at final follow-up

In this current follow-up, full medical records of all original participants were reviewed to obtain demographics, surgical data, and information from outpatient visits following surgery. Revision rates within the follow-up period due to implant failure were registered. Implant failure was defined as rod breakage, pseudarthrosis requiring revision, proximal junctional failure (PJF), or other (screw loosening, screw pullout, and dislodgement of cage).

All patients participating in the previous RCT and available for follow-up were invited to a clinical and radiographic examination a minimum of six years after the initial procedure. Patients unable to attend the clinical follow-up were retrospectively reviewed and reported on. Additionally, the patients were asked to respond to HRQoL Patient-Reported Outcome Measures (PROMs) using the validated questionnaires Scoliosis Research Society-22 revised (SRS-22r) [15] and European Quality of Life 5 Dimensions 3 Level (EQ-5D-3L) [16].

This study included both two-year and final follow-up results.

Radiographs

Full-spine radiographs were analyzed preoperatively, postoperatively, at two years, and at follow-up using the validated imaging software KEOPS (SMAIO, France) [17]. Pelvic incidence (PI), pelvic tilt (PT), sacral slope (SS), sagittal vertical axis (SVA), global lordosis (the largest lordotic angle between any two vertebrae), global kyphosis (the largest kyphotic angle between any two vertebrae), and L4-S1 (the angle between the upper endplate of L4 and the sacral endplate) were collected and reported for both groups. A radiographic assessment was performed by the primary author and not blinded to the primary intervention.

Statistics

All analyses were conducted using R version 4.2.0 (R Development Core Team, Vienna, Austria). Data was assessed for normality using histograms. Non-Gaussian data was reported as medians with interquartile ranges (IQRs) and tested using the Mann-Whitney U test. Categorical variables were reported as counts (%) and compared using Fisher's exact test. A significance level at <0.05 was set.

Ethical aspect and approval

The study was approved by the Data Protection Agency (j.nr. P-2021-763) and the National Committee on Health and Research Ethics (j.nr. H-21063169). Written informed consent was obtained from all participants in the clinical follow-up.

## Results

Pilot RCT results and two-year follow-up

Of the original 17 patients participating in the pilot RCT, the median age at the primary surgery was 67 (61-71) years, and 82% were female. The groups consisted of seven patients in the ALIF group and 10 in the non-ALIF group. Patients were comparable at baseline assessed on age, comorbidities, and radiological parameters pre- and postoperatively (Table 1, Table 2).

**Table 1:** Baseline patient characteristics in the total cohort stratified by intervention (N=17) Results are presented as medians (IQR) or counts (%). IQR: interquartile range, ALIF: anterior lumbar interbody fusion, CCI: Charlson Comorbidity Index

	ALIF (n=7)	Non-ALIF (n=10)	p-value
Age, years	65 (55-70)	67 (62-70)	0.540
Sex (female)	5 (71%)	9 (90%)	0.537
Follow-up time (years)	7.9 (6.5-9.1)	6.9 (5.0-8.6)	0.474
CCI	2 (1.5-3)	3 (2-3)	0.662
Instrumented levels	11 (10-13)	11 (9-14)	0.690
Three-column osteotomies	6 (85%)	6 (60%)	0.340
Smoking status			0.820
Current	2 (29%)	3 (30%)	
Previous	3 (42%)	2 (20%)	
Never	2 (29%)	5 (50%)	
Body mass index	22.3 (19.6-23.2)	25.5 (21.1-27.8)	0.220

**Table 2:** Radiographic parameters of the pilot RCT cohort stratified in groups by intervention (N=17) Values are reported as medians (IQR). RCT: randomized controlled trial, ALIF: anterior lumbar interbody fusion, IQR: interquartile range

	ALIF (n=7)	Non-ALIF (n=10)	p-value
Pelvic incidence (°)	49 (40-65)	44 (42-50)	0.740
Pelvic tilt (°)			
Preoperative	26 (17-30)	28 (23-28)	0.670
Postoperative	14 (13-23)	15 (10-19)	0.690
Sacral slope (°)			
Preoperative	29 (15-35)	20 (14-25)	0.241
Postoperative	31 (27-41)	28 (24-33)	0.315
Global lordosis (°)			
Preoperative	44 (38-54)	41 (24-44)	0.410
Postoperative	47 (45-64)	47 (45-52)	0.732
Global kyphosis (°)			
Preoperative	45 (37-60)	60 (48-67)	0.364
Postoperative	57 (50-60)	57 (47-69)	0.670
SVA (mm)			
Preoperative	87 (29-176)	79 (49-111)	0.812
Postoperative	31 (15-44)	52 (30-59)	0.270
L4-S1 (°)			
Preoperative	36 (34-42)	30 (24-40)	0.465
Postoperative	38 (28-43)	35 (32-43)	0.922

Two patients had died before the two-year follow-up, without undergoing revision surgery. No differences in revision rates between the supplemental ALIF and non-ALIF groups were found at two-year follow-up with 2/7 (29%) versus 3/10 (30%), respectively. Patients allocated to supplemental ALIF reported a lower HRQoL based on EQ-5D-3L with a median of 0.327 (0.25-0.49) and SRS-22r total score with a median of 2.9 (2.7-3.0) than the non-ALIF group with a median of 0.5 (0.45-0.59) and 4.1 (3.7-4.3) (p>0.05), respectively. All individual patient characteristics are displayed in the Appendices.

Current follow-up

From the 17 patients originally included in the pilot RCT, 15 were eligible for follow-up and 10 patients took part in the clinical examinations (Figure 1). This included six patients from the ALIF group and four from the non-ALIF group. Patients unable to attend the clinical follow-up were retrospectively reviewed. The median follow-up time was 7.7 (5.1-8.8) years. The overall time to failure for the total cohort was a median of 30 months. Stratified by intervention, time to failure was longer in ALIF patients with a median of 47 (28-53) months compared with non-ALIF patients with a median of 26 (9-31) months to failure. Individual patient characteristics are displayed in the Appendices.

Per protocol analysis showed a rate of revision among ALIF patients of three out of seven (43%) and eight out of 10 (80%) among non-ALIF patients. The most frequent reason for the revision was pseudarthrosis with rod breakage. No patients were revised due to other causes than implant-related. The cause of revision surgery stratified by intervention is displayed in Table 3.

**Table 3:** Revision causes in the total cohort (N=17) Eleven patients underwent revision. Values are reported as counts (%). ALIF: anterior lumbar interbody fusion, PJF: proximal junctional failure

	ALIF (n=7)	Non-ALIF (n=10)
Revision		
Pseudarthrosis	NA	1 (10%)
Pseudarthrosis with rod breakage	2 (29%)	5 (50%)
PJF	NA	2 (20%)
Screw breakage	1 (14%)	NA
No revision	4 (57%)	2 (20%)

Of the patients participating in the clinical follow-up, those receiving the supplemental ALIF reported a lower HRQoL score measured on the EQ-5D-3L with a median of 0.29 (0.17-0.38) and SRS-22r subscore with a median of 2.8 (2.5-2.9) than the no-ALIF with a median of 0.66 (0.51-0.78) and 4.1 (4.0-4.4), respectively.

Patient cases**

**Figure 2:**
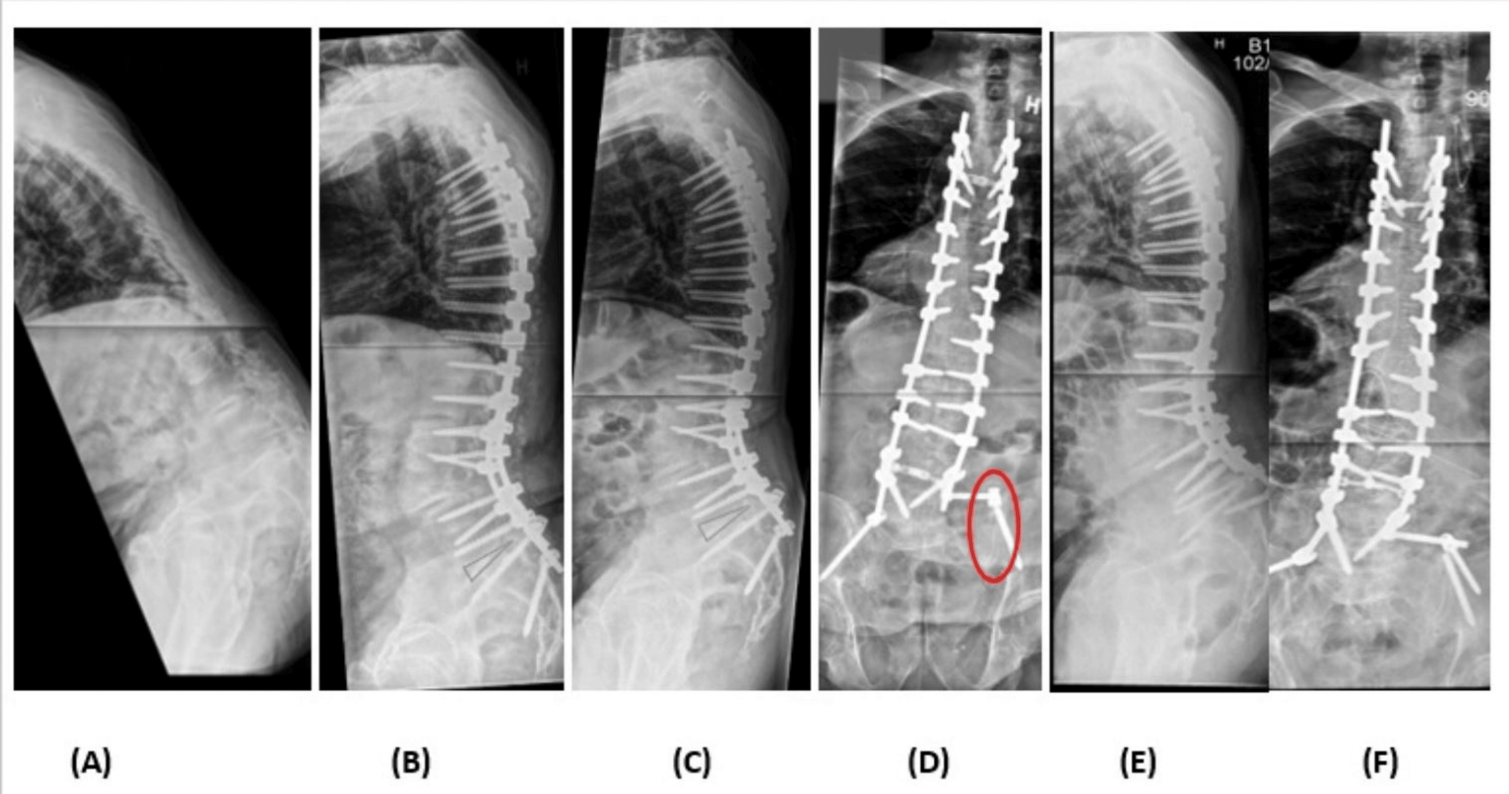
Case 1 A 71-year-old male was referred due to severe sagittal imbalance and back pain (A). He had previously undergone multiple decompression surgeries due to degenerative lumbar disease. At the time of referral, the patient presented with bilateral crista-costae collision, localized kyphosis in the lumbar region, and SVA of 285 mm. The patient underwent posterior instrumentation from Th4 to the pelvis, PSO at L3, and supplemental ALIF at L5/S1 (marked in blue), with an improvement of the sagittal imbalance (B). After 315 days, the patient was evaluated in the outpatient clinic due to acute back pain. CT scan revealed a broken iliac screw (red circle) and pseudarthrosis at the PSO level (C and D). The patient underwent revision surgery with replacement of both iliac screws, revision of pseudarthrosis at the PSO level, and additional SPO L1/L2 (E and F). SVA: sagittal vertical axis, PSO: pedicle subtraction osteotomy, ALIF: anterior lumbar interbody fusion, CT: computed tomography

**Figure 3:**
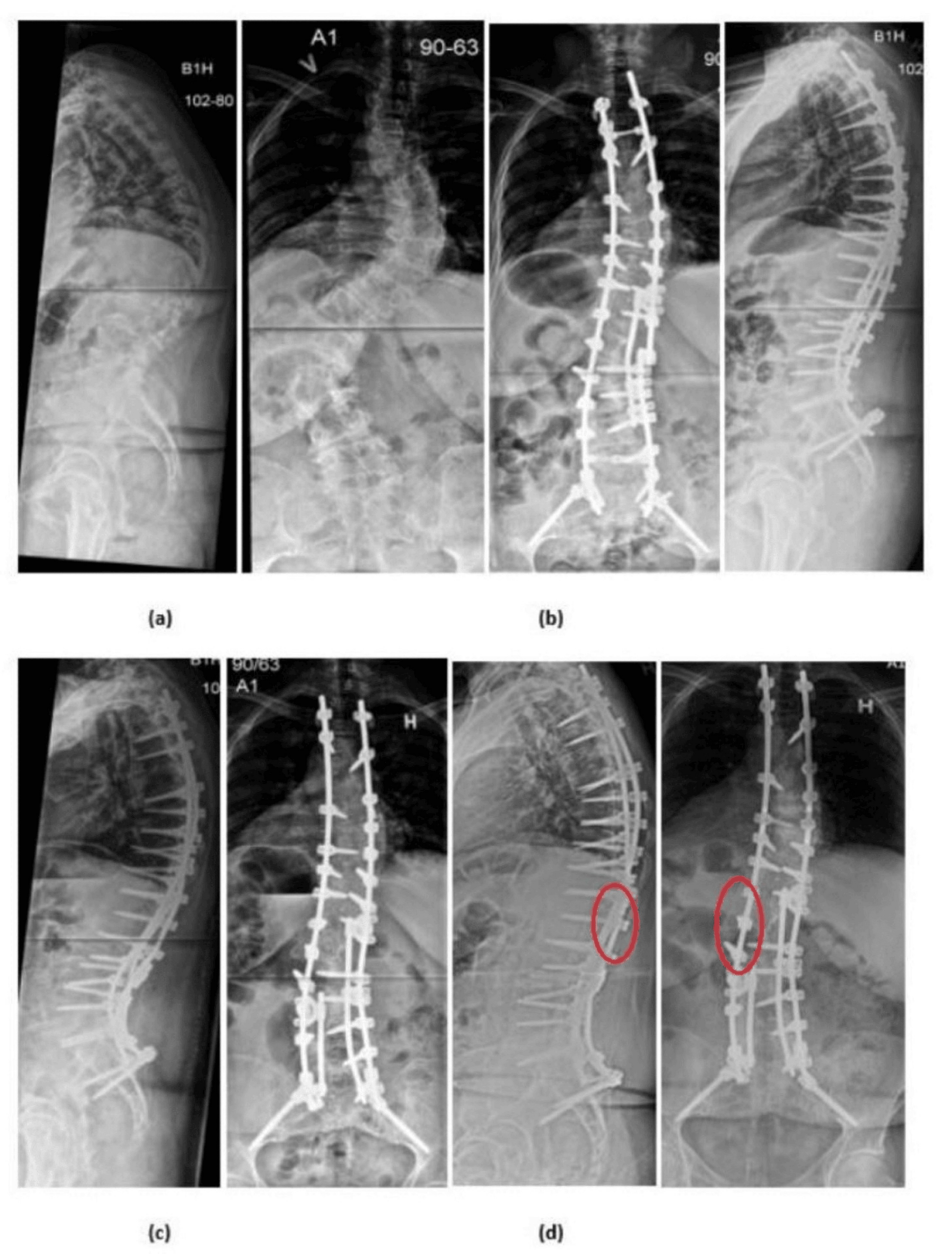
Case 2 A 53-year-old female was referred due to untreated thoracolumbar deformity known since childhood and progressing low back pain. She presented with kyphoscoliosis and a subjective feeling of positive sagittal imbalance (a). She underwent posterior instrumentation from Th2 to the pelvis with no supplemental ALIF (b). At 282 days postoperatively, the patient experienced acute onset of back pain and radiologically verified rod breakage due to pseudarthrosis. She underwent revision surgery with change of the rods and revision of the pseudarthrosis (c). At follow-up, the patient presented without pain and had returned to full-time employment. Radiographs showed rod breakage at L1, but with solid fusion and no loss of sagittal alignment, and thus was not scheduled for further revision (d). ALIF: anterior lumbar interbody fusion

Two detailed patient cases, each highlighting instances where revision surgery was required due to differing implant-related failures, are presented in Figure 2 (case 1) and Figure 3 (case 2).

## Discussion

In this study, we have investigated the long-term revision rates in patients with ASD undergoing posterior instrumentation and randomized to with or without supplemental ALIF. As revision rates remain high in the treatment of ASD [18-20], several methods have been investigated to lower the risk of additional surgery. Traditionally, ALIF was used in the treatment of degenerative spinal disease [21,22]. In recent years, ALIF has gradually become a part of the treatment in ASD as it facilitates lordosis correction, improves coronal curves, and possibly improves fusion rates [11,23,24]. With the properties that an additional ALIF can offer, the need for revision surgery could therefore be reduced. In this study, we did find a lower rate of revision surgery among patients undergoing supplemental ALIF compared to posterior instrumentation alone. This is in concordance with the results from a recent review by Burke et al. that found a lower risk of rod fractures in patients having received supplemental ALIF at the caudal end of the construct [19].

One of the most frequent causes of revision surgery in ASD patients is implant failure, often subsequent to pseudarthrosis [19,25,26]. Several factors play a role in the development of pseudarthrosis; hence, several considerations should be addressed during preoperative planning. Charosky et al. found an increased risk of mechanical failure in patients who underwent pedicle subtraction osteotomy (PSO) procedures and with no difference in revision rates among patients who had a combined anterior and posterior approach [8]. Moreover, Meyers et al. were unable to find an improved fusion rate and sagittal correction when adding an ALIF at the L5-S1 level [27]. Conversely, Dorward et al. found a 2% pseudarthrosis rate in long constructs at the level of the ALIF and an 11.9% pseudarthrosis rate at non-ALIF levels with a minimum follow-up time of two years [28]. Bae et al. found a lower rate of pseudarthrosis in patients undergoing lateral lumbar interbody fusion (LLIF) and ALIF in conjunction with posterior instrumentation (8.6% and 8.8%) compared with posterior instrumentation alone (15.3%), suggesting that supplemental ALIF could improve fusion rates [29]. In line with previous findings, the most frequent cause of revision in our study was pseudarthrosis, accounting for 7/11 (68%) of the revision cases, which occurred with a twofold incidence among non-ALIF patients. Due to a limited number of patients, we were not able to perform any sub-analyses on individual risk factors in this study. To accommodate the high number of mechanical failures leading to revision surgery in this cohort, changes in pelvic instrumentation have been gradually changed from traditional sacral instrumentation to sacral-alar-iliac instrumentation as proposed by Jain et al. since the pilot RCT was carried out [30].

The timespan from primary surgery to revision is of relevance. While many studies report one- or two-year revision rates, it has become evident that many complications occur at a later stage. Adogwa et al. reported that 50% of rod breakage following ASD surgery with supplemental interbody fusion occurs within the first three years of surgery and 30% occurs after five years [26]. Moreover, Lertudomphonwanit et al. reported a mean time to failure of 40 months [10]. Lastly, Charosky et al. reported a 48% reoperation risk at four years, increasing to nearly 80% at six-year follow-up when patients were fused to the sacrum [8]. Likewise, we saw an 80% failure rate within the eight-year period our patients were followed in patients with sacral fusion and non-ALIF, whereas patients fused to the sacrum with supplemental ALIF displayed a failure rate of 42%, suggesting an improved outcome for the latter. Moreover, we found that time to failure was dependent on the procedure performed, with a median time to failure of >2 years postoperatively. In light of these findings, studies with longer follow-up are warranted than the generally accepted two years.

Improvement of quality of life is one of the main goals of the surgical treatment. In this study, patients with supplemental ALIF reported a lower quality of life based on SRS-22r and EQ-5D than patients without ALIF both pre- and postoperatively. Although a small improvement in the SRS-22r and only a small decrease in the EQ-5D-3L was observed in the ALIF group, a substantially larger improvement was seen in patients with non-ALIF. Bae et al. reported a general improvement over time with no differences between patients who had undergone posterior instrumentation with supplemental ALIF and posterior instrumentation alone [29]. Due to the sample size in this study, it was not possible to elucidate the reason for the differences between the groups, and further research on HRQoL following these procedures is encouraged.

There are several limitations to this study. The small sample size did not allow us to make any conclusions on the effect of supplemental ALIF on revision surgery. With the limited sample size, the inherent risk of a type 2 error is present. This study can, therefore, only serve as a hypothesis-generating study for future research, and results should be interpreted with caution. It also highlights the general difficulties in performing an RCT study in this patient population. There is a limited population available for inclusion, especially when it comes to surgically naïve patients in ASD surgery. Of the 179 patients assessed for inclusion, only 38 were eligible for inclusion, and only about half of this population participated in the RCT study, underlining the difficulty in recruiting a sufficient number of participants to make conclusions on the results, which also led to the termination of the RCT study. This serves as a reminder that future randomized studies should broaden the inclusion criteria, increasing the external validity of the findings.

An alternative method of assessing failure, such as pseudarthrosis, could be CT evaluation of fusion masses in symptomatic patients. Although only 10/15 (66%) participated in the clinical follow-up, we were able to track all patients originally participating in the RCT and were, therefore, able to obtain long-term information on revision surgery and the cause of revisions on all original participants.

## Conclusions

A lower rate of revision surgery and a longer time to failure were found in patients undergoing ASD surgery with supplemental ALIF compared with posterior instrumentation alone. There is a need for additional research on larger populations to make conclusions on the effect of supplemental ALIF on posterior instrumentation alone in ASD surgery.

## Appendices

Individual patient characteristics are presented in Table 4.

**Table 4:** Individual patient characteristics of all patients (N=17) PSO: pedicle subtraction osteotomy, SPO: Smith-Petersen Osteotomy, VCR: vertebral column resection, CCI: Charlson Comorbidity Index, SRS-22R: Scoliosis Research Society-22 revised, ALIF: anterior lumbar interbody fusion, PJF: proximal junctional failure

Patient number	Intervention	Gender	Age at surgery, years	Instrumented levels	3-CO (PSO, SPO, and VCR)	CCI score	Time to revision, days	Revision cause	Follow-up time, years	SRS-22R subscore preoperative	SRS-22R subscore follow-up
1	No ALIF	Female	69	11	PSO	3	904	PJF	9.7	2.0	2.9
2	ALIF	Female	73	11	PSO	4	NA	NA	9.7	2.9	2.4
3	No ALIF	Male	61	14	PSO	0	3,016	Pseudarthrosis with rod breakage	9.6	3.4	4.1
4	ALIF	Female	47	15	NA	1	1,820	Pseudarthrosis with rod breakage	9.5	2.7	2.4
5	ALIF	Male	72	12	PSO	3	315	Iliac screw breakage and pseudarthrosis at PSO level	8.8	1.9	2.6
6	No ALIF	Female	53	15	NA	1	282	Pseudarthrosis with rod breakage	7.7	2.7	4.9
7	ALIF	Female	68	10	PSO	2	1,426	Pseudarthrosis with rod breakage	6.7	2.6	2.9
8	No ALIF	Female	59	10	NA	1	694	Pseudarthrosis with rod breakage	8.7	2.2	4.2
9	ALIF	Female	62	15	VCR	1	NA	NA	6.3	NA	3.1
10	ALIF	Female	65	9	SPO	3	NA	NA	7.9	2.6	3.5
11	No ALIF	Female	71	11	NA	3	NA	NA	0.1	1.9	NA
12	ALIF	Male	40	10	PSO	2	NA	NA	1.8	3.7	NA
13	No ALIF	Female	75	14	PSO	4	NA	NA	2.6	2.9	NA
14	No ALIF	Female	77	9	PSO	4	898	Pseudarthrosis without rod breakage	6.0	2.2	NA
15	No ALIF	Female	67	14	NA	2	1,072	Pseudarthrosis with rod breakage	5.0	1.7	NA
16	No ALIF	Female	67	8	SPO	3	252	Pseudarthrosis with rod breakage	8.5	1.7	NA
17	No ALIF	Female	66	8	PSO	2	9	PJF	9	2.05	NA

## References

[1] Faraj SS, van Hooff ML, Holewijn RM, Polly DW Jr, Haanstra TM, de Kleuver M (2017). Measuring outcomes in adult spinal deformity surgery: a systematic review to identify current strengths, weaknesses and gaps in patient-reported outcome measures. Eur Spine J.

[2] Pellisé F, Vila-Casademunt A, Ferrer M (2015). Impact on health related quality of life of adult spinal deformity (ASD) compared with other chronic conditions. Eur Spine J.

[3] Sullivan BT, Jain A, Aziz KT, Khanna AJ (2017). Clinical and radiographic evaluation of adult spinal deformities. Semin Spine Surg.

[4] Ames CP, Scheer JK, Lafage V (2016). Adult spinal deformity: epidemiology, health impact, evaluation, and management. Spine Deform.

[5] Schwab FJ, Blondel B, Bess S (2013). Radiographical spinopelvic parameters and disability in the setting of adult spinal deformity: a prospective multicenter analysis. Spine (Phila Pa 1976).

[6] Bari TJ, Hansen LV, Gehrchen M (2020). Surgical correction of adult spinal deformity in accordance to the Roussouly classification: effect on postoperative mechanical complications. Spine Deform.

[7] Yadla S, Maltenfort MG, Ratliff JK, Harrop JS (2010). Adult scoliosis surgery outcomes: a systematic review. Neurosurg Focus.

[8] Charosky S, Guigui P, Blamoutier A, Roussouly P, Chopin D (2012). Complications and risk factors of primary adult scoliosis surgery: a multicenter study of 306 patients. Spine (Phila Pa 1976).

[9] Sciubba DM, Yurter A, Smith JS (2015). A comprehensive review of complication rates after surgery for adult deformity: a reference for informed consent. Spine Deform.

[10] Lertudomphonwanit T, Bridwell KH, Kelly MP, Punyarat P, Theologis A, Sides BA, Gupta MC (2020). Relationship of the character of rod fractures on outcomes following long thoracolumbar fusion to the sacrum for adult spinal deformity. Spine J.

[11] Lee KY, Lee JH, Kang KC, Shin SJ, Shin WJ, Im SK, Park JH (2020). Strategy for obtaining solid fusion at L5-S1 in adult spinal deformity: risk factor analysis for nonunion at L5-S1. J Neurosurg Spine.

[12] Schroeder GD, Kepler CK, Millhouse PW, Fleischman AN, Maltenfort MG, Bateman DK, Vaccaro AR (2016). L5/S1 fusion rates in degenerative spine surgery: a systematic review comparing ALIF, TLIF, and axial interbody arthrodesis. Clin Spine Surg.

[13] Phan K, Thayaparan GK, Mobbs RJ (2015). Anterior lumbar interbody fusion versus transforaminal lumbar interbody fusion--systematic review and meta-analysis. Br J Neurosurg.

[14] Mobbs RJ, Phan K, Daly D, Rao PJ, Lennox A (2016). Approach-related complications of anterior lumbar interbody fusion: results of a combined spine and vascular surgical team. Global Spine J.

[15] Baldus C, Bridwell KH, Harrast J (2008). Age-gender matched comparison of SRS instrument scores between adult deformity and normal adults: are all SRS domains disease specific?. Spine (Phila Pa 1976).

[16] Cheung PW, Wong CK, Lau ST, Cheung JP (2018). Responsiveness of the EuroQoL 5-dimension (EQ-5D) in adolescent idiopathic scoliosis. Eur Spine J.

[17] Maillot C, Ferrero E, Fort D, Heyberger C, Le Huec JC (2015). Reproducibility and repeatability of a new computerized software for sagittal spinopelvic and scoliosis curvature radiologic measurements: Keops(®). Eur Spine J.

[18] Bari TJ, Hallager DW, Hansen LV, Dahl B, Gehrchen M (2021). Mechanical revision following pedicle subtraction osteotomy: a competing risk survival analysis in 171 consecutive adult spinal deformity patients. Spine Deform.

[19] Burke JF, Scheer JK, Lau D (2022). Failure in adult spinal deformity surgery: a comprehensive review of current rates, mechanisms, and prevention strategies. Spine (Phila Pa 1976).

[20] Hallager DW, Karstensen S, Bukhari N, Gehrchen M, Dahl B (2017). Radiographic predictors for mechanical failure after adult spinal deformity surgery: a retrospective cohort study in 138 patients. Spine (Phila Pa 1976).

[21] Mobbs RJ, Phan K, Malham G, Seex K, Rao PJ (2015). Lumbar interbody fusion: techniques, indications and comparison of interbody fusion options including PLIF, TLIF, MI-TLIF, OLIF/ATP, LLIF and ALIF. J Spine Surg.

[22] Mayer HM (2000). The ALIF concept. Eur Spine J.

[23] Geddes B, Glassman SD, Mkorombindo T, Gardner JQ, Carreon LY (2021). Improvement of coronal alignment in fractional low lumbar curves with the use of anterior interbody devices. Spine Deform.

[24] Park SJ, Park JS, Lee CS, Lee KH (2021). Metal failure and nonunion at L5-S1 after long instrumented fusion distal to pelvis for adult spinal deformity: anterior versus transforaminal interbody fusion. J Orthop Surg (Hong Kong).

[25] Smith JS, Klineberg E, Lafage V (2016). Prospective multicenter assessment of perioperative and minimum 2-year postoperative complication rates associated with adult spinal deformity surgery. J Neurosurg Spine.

[26] Adogwa O, Buchowski JM, Lenke LG (2020). Comparison of rod fracture rates in long spinal deformity constructs after transforaminal versus anterior lumbar interbody fusions: a single-institution analysis. J Neurosurg Spine.

[27] Meyers AJ, Wick JB, Rodnoi P, Khan A, Klineberg EO (2021). Does L5-S1 anterior lumbar interbody fusion improve sagittal alignment or fusion rates in long segment fusion for adult spinal deformity?. Global Spine J.

[28] Dorward IG, Lenke LG, Bridwell KH (2013). Transforaminal versus anterior lumbar interbody fusion in long deformity constructs: a matched cohort analysis. Spine (Phila Pa 1976).

[29] Bae J, Theologis AA, Strom R (2018). Comparative analysis of 3 surgical strategies for adult spinal deformity with mild to moderate sagittal imbalance. J Neurosurg Spine.

[30] Jain A, Brooks JT, Kebaish KM, Sponseller PD (2016). Sacral alar iliac fixation for spine deformity. JBJS Essent Surg Tech.

